# Magnetic molecularly imprinted polymers for the detection of aminopyralid in milk using dispersive solid-phase extraction†

**DOI:** 10.1039/c9ra05782j

**Published:** 2019-09-23

**Authors:** Yahui He, Sijia Tan, A. M. Abd EI-Aty, Ahmet Hacımüftüoğlu, Yongxin She

**Affiliations:** Beijing Technology and Business University 100048 P. R. China hyh@btbu.edu.cn +86-1068985456 +86-1068985456; Institute of Quality Standards & Testing Technology for Agro-Products, Chinese Academy of Agricultural Sciences Beijing 100081 P. R. China sheyongxin@caas.cn +86-1082106567 +86-1082106513; Department of Pharmacology, Faculty of Veterinary Medicine, Cairo University 12211-Giza Egypt; Department of Medical Pharmacology, Medical Faculty, Ataturk University 25240-Erzurum Turkey

## Abstract

A method for dummy molecular imprinting-magnetic dispersive solid-phase extraction (MI-MDSPE) coupled with liquid chromatography-tandem mass spectrometry (LC-MS/MS) was developed for the selective determination of aminopyralid in milk. The magnetic material and polymers were combined *via* a series of modifications in Fe_3_O_4_. Fe_3_O_4_@SiO_2_–NH_2_@MIP, Fe_3_O_4_@SiO_2_–COOH@MIP and two types of aminopyralid-specific magnetic molecularly imprinted polymers (MMIPs) were prepared on the surface of magnetic nanoparticles modified with amino and carboxyl groups. The morphology and magnetic properties of the polymer were characterized. Fe_3_O_4_@SiO_2_–NH_2_@MIP exhibits not only good dispersibility and magnetic properties, but also an outstanding recognition pattern to the target analyte. Adsorption experiments demonstrated that Fe_3_O_4_@SiO_2_–NH_2_@MIP, with a high specific surface area and fast mass transfer rate, had a higher affinity than Fe_3_O_4_@SiO_2_–COOH@MIP towards aminopyralid. Under the optimized MI-MDSPE conditions, the method had good linearity (*R*^2^ > 0.9972), excellent recoveries (83.3–90%), and good precision (relative standard deviations (RSDs) < 12.6%). This method has limits of detection (LOD) and quantification (LOQ) of 0.231 and 0.77 μg kg^−1^, respectively, indicating that these MMIPs can be used to analyse trace levels of aminopyralid in real samples.

## Abstract 2

(a) Preparation of Fe_3_O_4_@SiO_2_–NH_2_@MMIP. (b) Diagram of magnetic molecularly imprinted polymers for the detection of aminopyralid in milk using dispersive solid-phase extraction.

## Graphical abstract

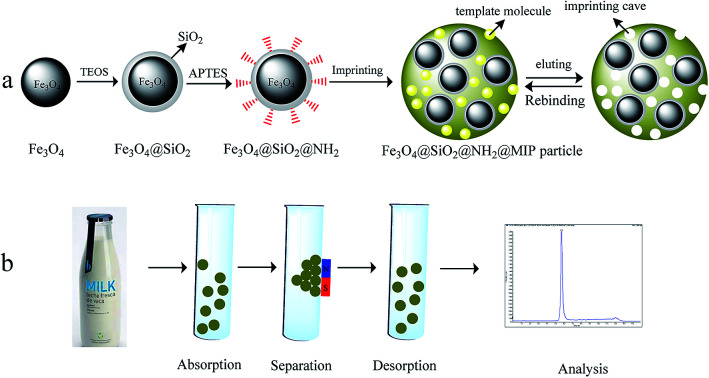

## 1. Introduction

Aminopyralid is a synthetic auxin herbicide with a pyridine ring and carboxylic acid functional group. The mechanism of action of this synthetic hormone herbicide is similar to that of a growth hormone.^1^ Aminopyralid exerts highly efficient and selective weed removal since it can be quickly absorbed by the stems and leaves of plants and interrupt plant growth, causing rapid death.^2,3^ Despite its low toxicity and high herbicidal activity, concerns have been raised^4^ because its improper use may cause negative impacts on human health. Research has shown that aminopyralid taken up by livestock through animal feed causes irreversible damage to human kidneys, because it exists as an exogenous anti-nutritional factor in the animal-derived foods.^5^ In China, standard rule GB 2763-2016 stipulates a maximum residue limit (MRL) of only 0.1 mg kg^−1^ for aminopyralid in barley, wheat, oats, and triticale, and there are no relevant limits set for animal-derived foods. Considering the low MRL of aminopyralid in milk, 0.02 mg kg^−1^, set by the Codex Alimentarius Commission (CAC), an efficient pre-treatment method and detection protocol for this herbicide in milk is of great importance.^6^

The analytical methodologies applied for the determination of aminopyralid in different matrices has been reviewed. For instance, liquid chromatography-tandem mass spectrometry (LC-MS/MS)^7^ and gas chromatography coupled with electron capture detection (GC-ECD)^8^ have been developed for determination of aminopyralid in vegetables and soil. Traditional sample pretreatment procedures, solid-phase extraction (SPE), and quick, easy, cheap, effective, rugged, and safe “QuEChERS” method were used in association with these chromatographic methods. However, these pretreatment methods have drawbacks of low selectivity and adsorption capacity, resulting in the inability to enrich trace amounts of target analytes from complex matrices.

As a promising separation technology, molecular imprinting techniques (MITs) involve predetermined molecular structures and specific recognition. Molecularly imprinted polymers (MIPs) with specific cavity structures are developed based on “the antigen and antibody” and “the lock and key” hypotheses.^9^ MIPs are tailored according to the target molecules. Advantages such as good stability, strong affinity, high selectivity, and low costs enable MIPs to be used in a wide range of applications in agriculture, environmental analysis, medicine, and other areas.^10^

Two disadvantages of MIPs are that the template molecules cannot be completely cleaned and the mass transfer resistance is high; these issues have substantially reduced the amount of polymer that can be adsorbed.^11^ Molecular imprinting using a dummy template that is similar in structure to the target molecules has eliminated false positive results caused by template leakage.^12^ Moreover, magnetic molecular imprinting has demonstrable advantages for sample pretreatment, such as a large specific surface area, low resistance to mass transfer, and high separation speed.^13^ Magnetic nanoparticles (MNPs) have the unique properties of nanocrystals, such as general small size effects, superparamagnetism, and surface effects,^14^ and can be modified by active functional groups, such as –COOH, –OH, and –NH_2_, to enable facile combination with MITs for use in sample pretreatment. However, MNPs exhibit a strong aggregation tendency and are easily oxidized in air, so materials including silica, surfactants, octadecylsilane, and others have been used to modify MNPs to overcome these shortcomings.^15,16^ Magnetic molecularly imprinted polymers (MMIPs) are prepared on the surface of a magnetic carrier using surface imprinting to distribute the imprinted sites on the surface of the MIP.^17,18^

Magnetic dispersive solid-phase extraction (MDSPE) based on separation with a magnetic sorbent and an external magnetic field can eliminate the need for a centrifugation step, shorten the separation time for the target molecules from solution, and reduce the loss of liquid and solid during the separation.^19–21^ In molecular imprinting-magnetic dispersive solid-phase extraction (MI-MDSPE), a MIP is synthesized on the surface of functionalized MNPs, which are then added to sample matrices containing target analytes for clean-up. After shaking the mixture, the MMIP that adsorbs the target substance is isolated from the solution using an external magnetic field.^22,23^ Compared with other pretreatment methods, MI-MDSPE combines the quick separation of magnetic particles and special selectivity of MIPs and has high specificity and simple method procedures.

In the present study, a novel MIP using picloram as a dummy template was successfully imprinted on the surface of Fe_3_O_4_@SiO_2_–NH_2_ for the specific adsorption of aminopyralid. The characteristics and binding tendency of the MMIP were investigated. By optimizing a range of conditions for MI-MDSPE, the MMIP was applied for the determination of aminopyralid in milk by dispersive SPE coupled with LC-MS/MS.

## 2. Experimental

### 2.1 Chemicals and reagents

Aminopyralid and picloram (purity > 99%) were obtained from Dr Ehrenstorfer GmbH (Augsburg, Germany). 4-Vinylpyridine (4-VP) was purchased from Alfa Aesar (Massachusetts, USA). Trimethylolpropane trimethacrylate (TRIM), 1-ethyl-3-(3-dimethylaminopropyl)carbodiimide (EDC), and *N*-hydroxysuccinimide (NHS) were procured from Sigma-Aldrich (St. Louis, CA, USA). 2-2′-Azobisisobutyronitrile (AIBN) was acquired from Merck (Darmstadt, Germany). Iron(ii) chloride tetrahydrate (FeCl_2_·4H_2_O), iron(iii) chloride hexahydrate (FeCl_3_·6H_2_O), tetraethyl orthosilicate (TEOS), (3-aminopropyl)triethoxysilane (APTES), poly(ethylene glycol)bis(carboxymethyl) ether, and ammonium hydroxide were purchased from Beijing Chemical Co. (Beijing, China). Methanol (MeOH), acetonitrile (MeCN), *n*-hexane, and formic acid were of HPLC grade and supplied by Thermo Fisher Scientific (Waltham, MA).

### 2.2. Instrumentation

Milk samples were analysed with an Agilent HPLC system (1200 series, Agilent Technologies, USA) coupled with a triple quadrupole mass spectrometer (AB2000, AB SCIEX, USA) equipped with an electrospray ionization (ESI) source. HPLC analysis was performed on a Waters 2695 Alliance HPLC system (Waters Corporation, Milford, MA, USA) equipped with a diode array detector (DAD). X-ray diffraction (XRD) measurements used to determine the crystal structure of the nanoparticles were performed on a D-Max 2200 VPC diffractometer (Rigaku, Tokyo, Japan). Fourier transform infrared (FT-IR) spectroscopy (Philips PU9800, Philips Analytical, Cambridge, UK) was used to characterize the polymers. The hysteresis loop of magnetic compounds was measured using a vibrating sample magnetometer (VSM, LakeShore 7407, OH, USA).

### 2.3. Chromatographic conditions

HPLC and LC-MS/MS were used to determine the adsorption performance of the MIPs and the amount of aminopyralid in the milk samples, respectively. In HPLC, aminopyralid was separated on a Waters Sunfire C_18_ column (4.6 × 150 mm, particle size of 5 μm). The mobile phase consisted of 0.1% formic acid in ultrapure water (A) and methanol (B). Gradient elution was carried as follows: 10% B, held for 1 min, increased to 80% B in 7 min, held for 1 min, decreased to 10% in 8.1 min, and held for 1.9 min to equilibrate the column. The flow rate of the mobile phase was maintained at 1 mL min^−1^ and the column temperature was set at 28 °C. The injection volume was 20 μL.

The analytical column for LC-MS/MS was a Waters XSelect HSS T3 column (2.1 × 150 mm, particle size of 5 μm). The mobile phase was composed of ultrapure water (A) and methanol (B) with a flow rate of 0.3 mL min^−1^. Gradient elution was carried out as follows: increased from 40% to 60% B in 2 min, increased to 90% B in 8 min, held for 2 min, decreased to 40% B in 10.1 min, and held for 4.9 min to equilibrate the column. The injection volume was 5 μL.

### 2.4. MS/MS conditions

A mass spectrometer equipped with an ESI source was operated in multiple reaction monitoring (MRM) mode to determine aminopyralid in positive ionization mode. The conditions of the ESI source were as follows: the ion source temperature was 450 °C, and the gas pressures of the collision-activated dissociation (CAD), curtain gas (CUR), ion source gas 1 (GS1), and ion source gas 2 (GS2) were 8, 40, 50, and 50 psi, respectively. The qualitative ion pair (*m*/*z*) consisted of 207.0 and 189, and the quantitative ion pair (*m*/*z*) consisted of 207.0 and 161. The declustering potential (V) and collision energy (eV) were 39.29, 29.2, and 18.41, respectively.

### 2.5. Synthesis and modification of Fe_3_O_4_ magnetic nanoparticles

The magnetic Fe_3_O_4_ nanoparticles were synthesized by coprecipitation.^24^ Briefly, FeCl_2_·4H_2_O (0.86 g) and FeCl_3_·6H_2_O (2.35 g) were dissolved in 100 mL of ultrapure water in a three-neck flask and vigorously stirred under a nitrogen atmosphere at 60–70 °C. As the temperature was elevated to 80 °C, 10 mL of a 25% ammonia solution was added, and the mixture was stirred vigorously for 30 min. Then, 0.1 g of sodium citrate was added to increase the dispersion of the nanoparticles, and stirring was continued for another 30 min. After finishing the reaction, the mixture was cooled to room temperature, and then the black magnetic precipitates were isolated from the solvent by a permanent magnet and washed several times alternating between deionized water and ethanol until the pH of the eluent approached neutral.

The Fe_3_O_4_@SiO_2_ nanocomposites were synthesized according to the hydrolysis of a silylation reagent as follows. The prepared Fe_3_O_4_ (approximately 1 g) was dispersed in 50 mL of ethanol *via* ultrasound. Then, 25 mL (approximately 0.5 g of Fe_3_O_4_) of the solution of the magnetic nanoparticles dissolved in ethanol was measured and separated by magnet. After discarding the ethanol supernatant, 20 mL of double-distilled water and 100 mL of ethanol were added to the solution and sonicated for 30 min. Afterward, 1 mL of ammonium hydroxide (25%, w/w) and 2 mL of TEOS were added to the mixture in a dropwise manner. The mixture was stirred at room temperature for 24 h.

The Fe_3_O_4_@SiO_2_ particles were modified by amino groups. Briefly, 1.5 mL of APTES was added to the above Fe_3_O_4_@SiO_2_ reaction solution and stirred. The stirring continued at 40 °C for 24 h. The obtained Fe_3_O_4_@SiO_2_–NH_2_ was collected by a magnet and thoroughly washed with ethanol several times.

Similar to the production of Fe_3_O_4_@SiO_2_–NH_2,_ carboxyl groups were grafted on the Fe_3_O_4_@SiO_2_ nanocomposites. The Fe_3_O_4_@SiO_2_–NH_2_ nanoparticles were dispersed in 0.01 mmol L^−1^ PBS buffer with a pH of 7.4. After being completely dissolved, 80 μL of poly(ethylene glycol)bis(carboxymethyl) ether, 5 mL of 10 mg mL^−1^ EDC, and 5 mL of 10 mg mL^−1^ NHS were added. Subsequently, the obtained dispersion was mechanically stirred at room temperature for 8 h, and the precipitate was collected by a magnet, repeatedly washed with ethanol, and dried in a vacuum.

### 2.6. Procedures for the preparation of MMIPs

A series of polymers were prepared to investigate the effect of adsorption amount and polymerization ratio of the functional monomer and crossing-liker as shown in Table S1.† The results showed that the optimal molar ratio of template to functional monomer and cross-linker was 1 : 6 : 6. The synthesis of MIPs with a molar ratio of picloram/4-VP/TRIM = 1 : 6 : 6 is described as follows. The dummy template picloram (0.2 mmol) was dissolved in 30 mL of methanol, and then the functional monomer 4-VP (1.2 mmol) was added. This mixture was stirred for 30 min on a shaker table to form the template–monomer complex. Then, Fe_3_O_4_@SiO_2_–NH_2_ (100 mg) dissolved in 10 mL of methanol, the cross-linker TRIM (1.2 mmol) and the initiator AIBN (50.0 mg) were added to the above mixture solution and ultrasonically mixed for 5 min. The reaction mixture was purged with nitrogen and stirred at 60 °C for 24 h. After polymerization, the polymers were collected magnetically and washed with methanol/acetic acid (80 : 20, v/v) until no precursor was detected by HPLC. Finally, the MMIPs were rinsed with methanol until neutral pH was achieved and dried under vacuum at 50 °C for 24 h.

Magnetic nonimprinted polymers (MNIPs) were synthesized using the same method as above, except the template picloram was omitted.

For comparison, Fe_3_O_4_@SiO_2_–COOH@MIP was prepared by the same procedure, except Fe_3_O_4_@SiO_2_–COOH replaced Fe_3_O_4_@SiO_2_–NH_2_ in the polymerization process.

### 2.7. Measurement of kinetic adsorption and adsorption isotherm curves

Dynamic and static equilibrium adsorption experiments were conducted to evaluate the adsorption capacity of different polymers.^25^ Ten milligrams each of MMIP or MNIP was dispersed in a 2 mL centrifuge tube containing 1 mL of methanol solution with aminopyralid at 15 μg mL^−1^ and shaken for different times (0.5, 1, 1.5, 2, 4, 6, and 8 h). Similarly, for the static experiments, 10 mg of MMIP or MNIP was mixed with 1 mL of aminopyralid in methanol at various concentrations (5, 10, 15, 20, 30, 50, 100, and 150 μg mL^−1^). After shaking for 1 h, the samples were collected by a magnet. The residual aminopyralid in the supernatant was detected by HPLC. The amount of aminopyralid adsorbed on the MMIP and MNIP particles (*Q*, μg g^−1^) was calculated by eqn (1):

1 *Q* = (*C*_o_ − *C*_e_)*V*/*m*

where *C*_o_ (μg mL^−1^) and *C*_e_ (μg mL^−1^) represent the target concentration in the supernatant initially and at equilibrium, respectively. *V* (mL) is the solution volume, and *m* (mg) is the mass of MMIP or MNIP particles.

The subsequent Scatchard analysis of the MMIP was evaluated. The Scatchard curve was plotted using the Scatchard equation, *Q*/*C*_e_ = (*Q*_max_ − *Q*)/*K*_d_, and the dissociation constant (*K*_d_) and the maximum absorption of binding sites (*Q*_max_) were calculated.

The selective adsorption of the MMIP was evaluated using chloramphenicol as the target with the same method.

### 2.8. Optimization of MDSPE

Ten milligrams of Fe_3_O_4_@SiO_2_–NH_2_@MIP was added to a 5 mL centrifuge tube as a sorbent for dispersive SPE, and 1 mL of 50 μg L^−1^ aminopyralid solution was added to the tube and shaken to extract the target compound. Finally, the mixture was separated by a magnet, and the eluent was analysed using LC-MS/MS. Based on the analyte recovery, the extraction solution, extraction time, pH, elution solvent, and elution time were optimized.

### 2.9. Analysis of milk samples

Milk samples (2 g) spiked with aminopyralid standard solutions (10, 20, and 50 μg L^−1^) were first extracted using acetonitrile (10 mL) for 5 min. Anhydrous magnesium sulfate (4 g) and sodium chloride (1 g) were added to remove water from the milk sample. The solution was centrifuged at 5000 rpm for 10 min, and then 5 mL of the supernatant was transferred to a new centrifuge tube containing 2 mL of *n*-hexane saturated with acetonitrile to remove the upper fat layer. Fe_3_O_4_@SiO_2_–NH_2_@MIP (10.0 mg) was suspended in 1 mL of the above solution, and then the supernatants were adjusted to pH 5. After shaking for 10 min, the target was eluted using 3 mL of methanol for 5 min on a shaker table. The eluent was collected by a magnet and analysed using LC-MS/MS. Each sample was analysed in triplicate.

## 3. Results and discussion

### 3.1. Characterization of MMIPs and MNIPs

**Fig. 1:**
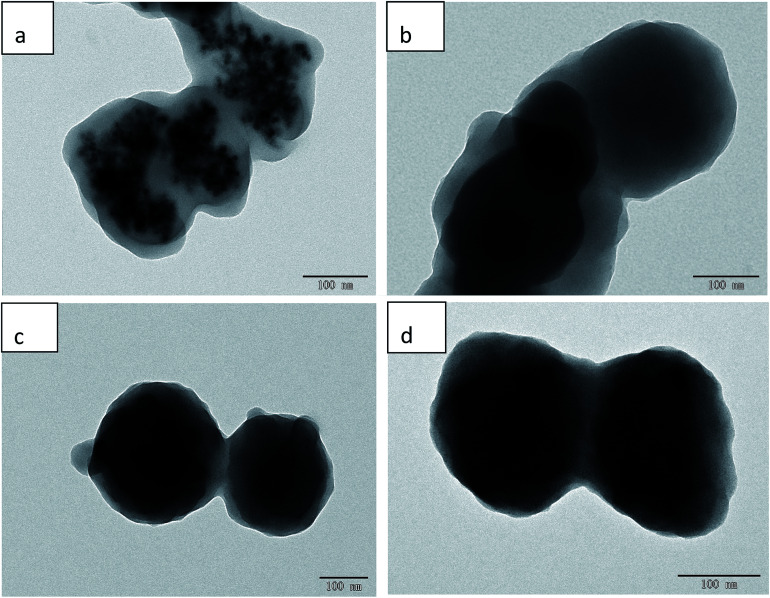
TEM images of Fe_3_O_4_@SiO_2_–NH@MIP (a), Fe_3_O_4_@SiO_2_–NH_2_@NIP (b), Fe_3_O_4_@SiO_2_–COOH@MIP (c), and Fe_3_O_4_@SiO_2_–COOH@NIP (d).

The prepared samples were characterized by transmission electron microscopy (TEM), FT-IR spectroscopy, XRD, and VSM. The particle size and morphology of the MMIPs and MNIPs can be clearly observed by TEM (Fig. 1). The TEM images of Fe_3_O_4_, Fe_3_O_4_@SiO_2_–COOH, and Fe_3_O_4_@SiO_2_–NH_2_@NIP are displayed in Fig. S1.† The Fe_3_O_4_ in Fe_3_O_4_@SiO_2_–COOH@MIP (Fig. 1c) and Fe_3_O_4_@SiO_2_–COOH@NIP (Fig. 1d) is seriously agglomerated, and isolated magnetic microspheres are completely invisible. However, although the Fe_3_O_4_ in MMIP modified with amino groups (Fig. 1a) is agglomerated, single magnetic microspheres can still be observed. Similar to the polymers modified with carboxyl groups, Fe_3_O_4_@SiO_2_–NH_2_@NIP (Fig. 1b) also has agglomerated Fe_3_O_4_. The prepared Fe_3_O_4_ particles are approximately 10 nm in diameter and the particle size increases 10-fold to 100 nm after molecular imprinting. This may indicate that Fe_3_O_4_@SiO_2_–NH_2_@MIP is more suitable than Fe_3_O_4_@SiO_2_–COOH@MIP for dispersive SPE.

**Fig. 2:**
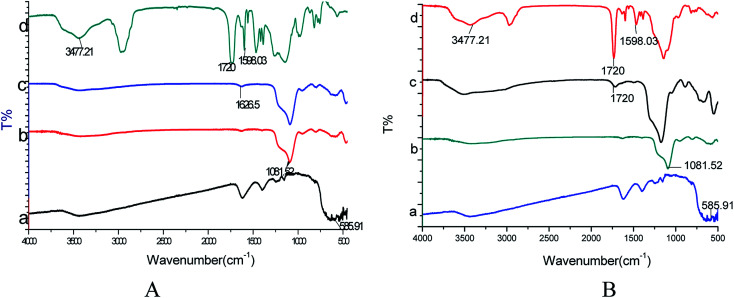
(A) FT-IR spectra of Fe_3_O_4_ (a), Fe_3_O_4_@SiO_2_ (b), Fe_3_O_4_@SiO_2_–NH_2_ (c) and Fe_3_O_4_@SiO_2_–NH_2_@MIP (d); (B) FT-IR spectra of Fe_3_O_4_ (a), Fe_3_O_4_@SiO_2_ (b), Fe_3_O_4_@SiO_2_–COOH (c), and Fe_3_O_4_@SiO_2_–COOH@MIP (d).

Although the black Fe_3_O_4_ particle is surrounded by a layer of grey, this does not mean that the grey layer is the molecularly imprinted layer; this layer may also be composed of SiO_2_. So further characterization is needed to show that the polymer has been successfully polymerized using FT-IR analysis (Fig. 2). The absorption peak at approximately 585.91 cm^−1^ is related to Fe–O vibrations (Fig. 2A(a) and B(a)). A strong characteristic Si–O stretching vibration peak at 1081.52 cm^−1^ appeared for Fe_3_O_4_@SiO_2_ (Fig. 2A(b) and B(b)), which indicated that SiO_2_ was successfully coating the surface of Fe_3_O_4_. The peaks at 1626.5 cm^−1^ and 1720 cm^−1^ were attributed to the stretching vibrations of the amino group in Fe_3_O_4_@SiO_2_–NH_2_ (Fig. 2A(c)) and of the carboxyl group in Fe_3_O_4_@SiO_2_–COOH (Fig. 2B(c)), respectively, indicating successful modification with NH_2_ and COOH groups on the Fe_3_O_4_@SiO_2_ surface. In the FT-IR spectra of Fe_3_O_4_@SiO_2_–NH_2_@MIP (Fig. 2A(d)) and Fe_3_O_4_@SiO_2_–COOH@MIP (Fig. 2B(d)), the absorption peaks of –OH groups at 3477.21 cm^−1^, C=O groups at 1720 cm^−1^ and C=C groups at 1598.03 cm^−1^ indicated the successful grafting of a polymer layer on the Fe_3_O_4_@SiO_2_–NH_2_ and Fe_3_O_4_@SiO_2_–COOH particles.

The XRD patterns of the nanoparticles are presented in Fig S2a and b.† The pattern of Fe_3_O_4_ has six characteristic peaks at 2*θ* = 30.18, 35.57, 43.16, 53.45, 57.04 and 62.67 corresponding to the (220), (311), (400), (422), (511) and (440) crystal faces,^26^ respectively. We can see that the intensities of the peaks are weakened, but the characteristic peak positions did not change, showing that Fe_3_O_4_@SiO_2_–NH_2_@MIP and Fe_3_O_4_@SiO_2_–COOH comprised Fe_3_O_4_ nanoparticles and that the XRD phase of Fe_3_O_4_ with an inverse spinel structure did not change during the synthesis.

**Fig. 3:**
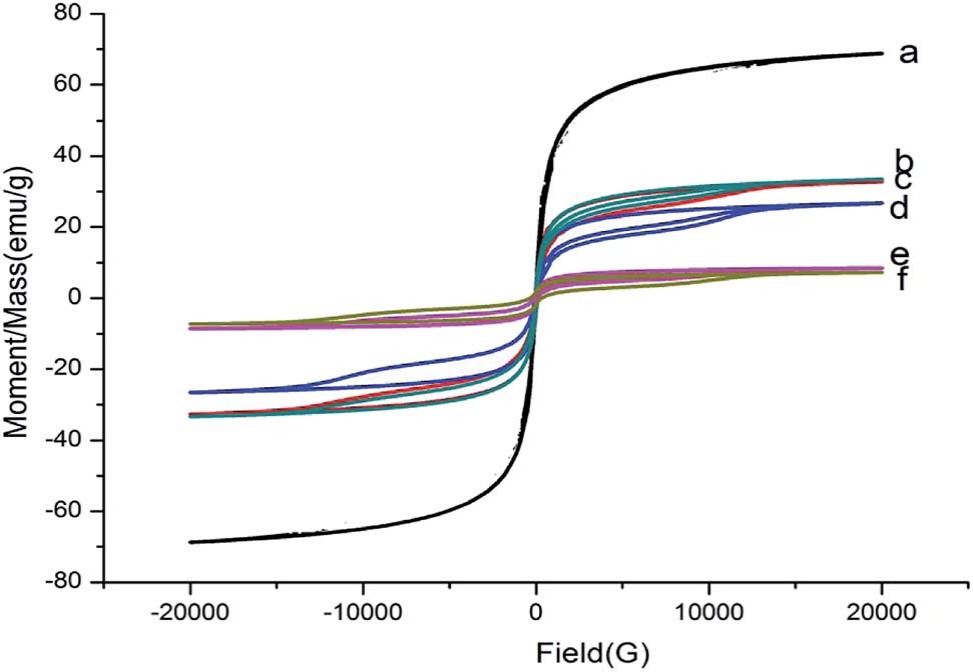
Hysteresis loop of Fe_3_O_4_ (a), Fe_3_O_4_@SiO_2_ (b), Fe_3_O_4_@SiO_2_–NH_2_ (c), Fe_3_O_4_@SiO_2_–COOH (d), Fe_3_O_4_@SiO_2_–NH_2_@MIP (e), Fe_3_O_4_@SiO_2_–COOH@MIP (f).

The magnetic properties of the synthesized particles were analysed by VSM, and the results are illustrated in Fig. 3. It is obvious that Fe_3_O_4_ showed no hysteresis, indicating that Fe_3_O_4_ was superparamagnetic.^27^ However, hysteresis was observed after coating with SiO_2_ and modifying with NH_2_ and COOH groups. The composite material can have a certain cohesive force, and the saturation magnetization of Fe_3_O_4_@SiO_2_, Fe_3_O_4_@SiO_2_–NH_2_, Fe_3_O_4_@SiO_2_–COOH, Fe_3_O_4_@SiO_2_–NH_2_@MIP and Fe_3_O_4_@SiO_2_–COOH@MIP gradually decreased from 72.67 to 36.96, 35.82, 29.01, 13.72, and 11.93 emu g^−1^, respectively. These results showed not only that the MMIPs were prepared successfully but also that the prepared polymers have good magnetic properties and can be applied in dispersive SPE for the rapid detection of compounds from solution.

### 3.2. Adsorption properties of the MMIP

**Fig. 4:**
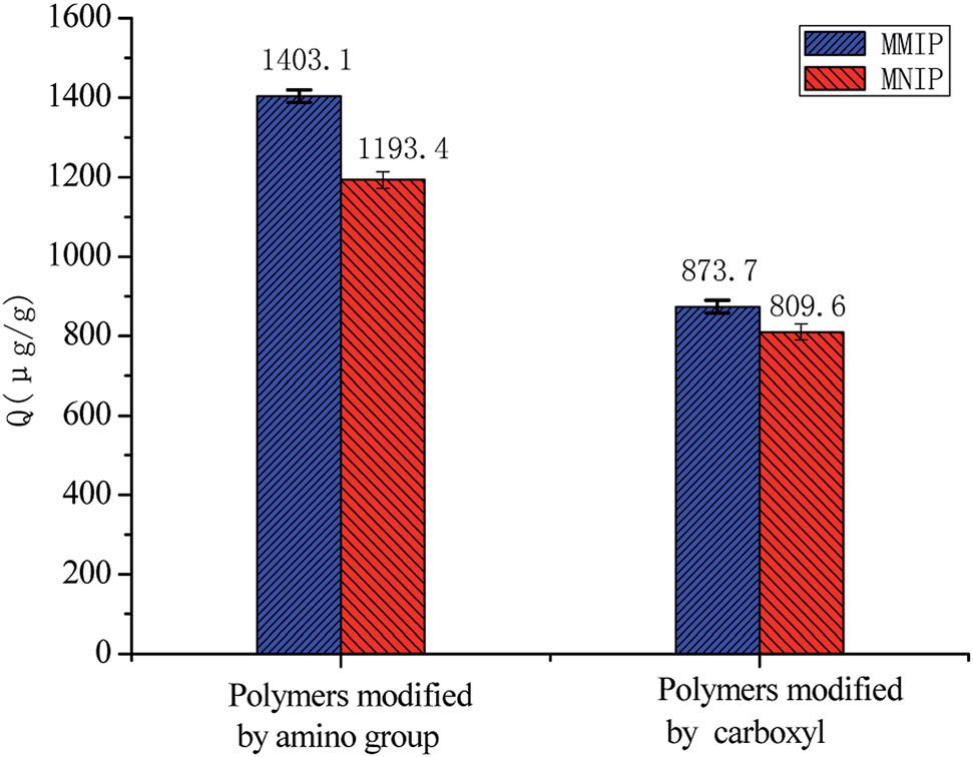
Comparison of the capacity of polymers modified by amino and carboxyl groups.

Fig. 4 shows the capacity of 10 mg of polymers modified by amino and carboxyl groups for adsorbing a 15 μg mL^−1^ aminopyralid solution in methanol. We observed that the amount adsorbed onto polymer surface modified by amino groups was much higher than that with carboxyl groups. This finding might be attributed to electrostatic interaction between MIP and template molecule modified by Fe_3_O_4_@SiO_2_–NH_2_ and Fe_3_O_4_@SiO_2_–COOH. Furthermore, the electrostatic interaction between MIP and template molecule modified by carboxyl was quite weak compared with amino. The adsorption capacity of the MMIP with different amounts of Fe_3_O_4_@SiO_2_–NH_2_ (50, 100, 150, and 200 mg) was evaluated. When 100 mg of Fe_3_O_4_@SiO_2_–NH_2_ was dispersed in a sample solution, the amount of aminopyralid adsorbed to the MMIP reached a maximum. Due to the occupation of the binding sites of the polymer, the adsorption decreases as the quantity of magnetic nanoparticles increases.

**Fig. 5:**
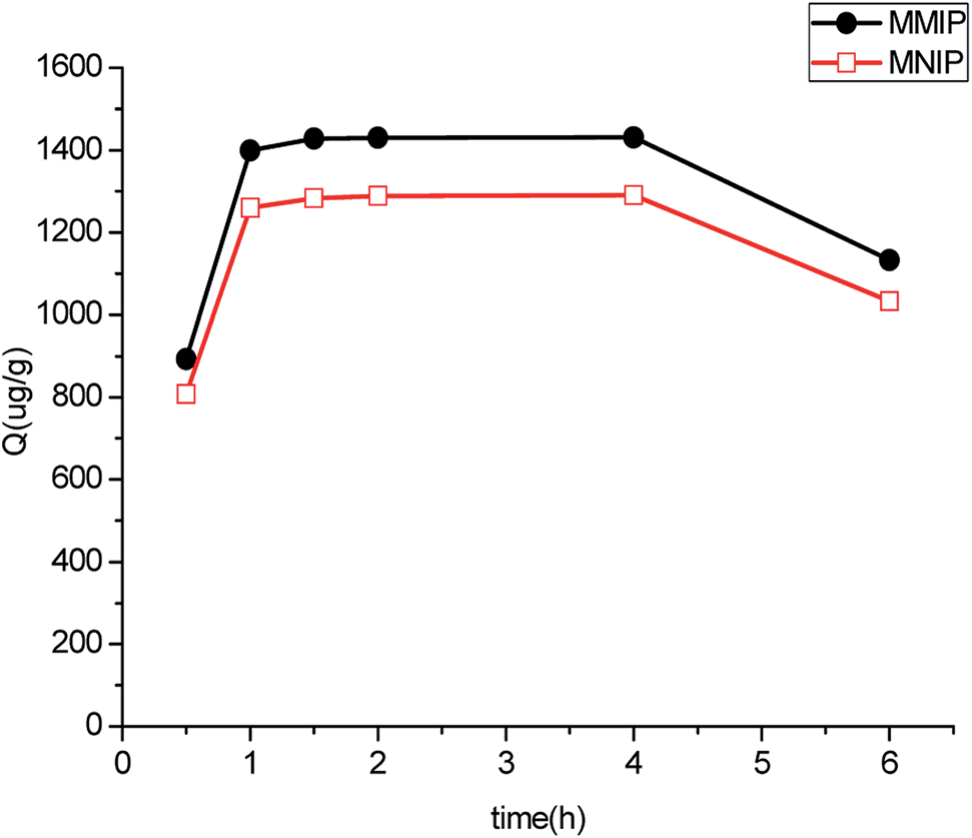
Kinetics adsorption curve of MMIP and MNIP.

Dynamic binding experiments were performed to determine the adsorption saturation time of polymers, and the results are shown in Fig. 5. The amount of aminopyralid adsorbed to Fe_3_O_4_@SiO_2_–NH_2_@MIP reached equilibrium at 1 h and desorption occurred when the shaking time exceeded 4 h, suggesting that the adsorption is fast.

**Fig. 6:**
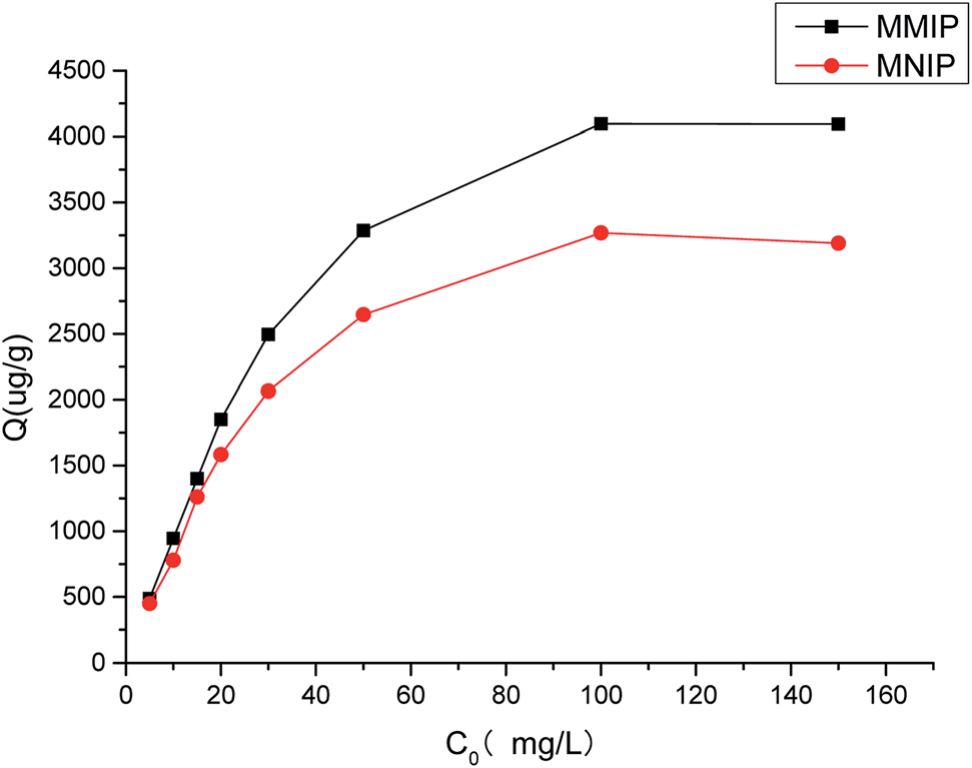
Isothermal adsorption curve of MMIP and MNIP.

Fig. 6 shows the static binding isotherms of aminopyralid on MMIP and MNIP. The adsorption capacities of the MMIP and MNIP increased with an increasing initial concentration of aminopyralid. The difference between the MMIP and MNIP at low aminopyralid concentrations was slight, but the absorption of aminopyralid on the MMIP was higher than that on the MNIP at aminopyralid concentrations over 10 mg L^−1^. This revealed that the MMIP showed a higher binding affinity for aminopyralid than the MNIP. Such a result may be due to the nonspecific adsorption caused by nonspecific recognition sites on the surface of the MNIP being important at low concentrations, while the specific binding sites of MMIP dominated at high concentrations.

**Fig. 7:**
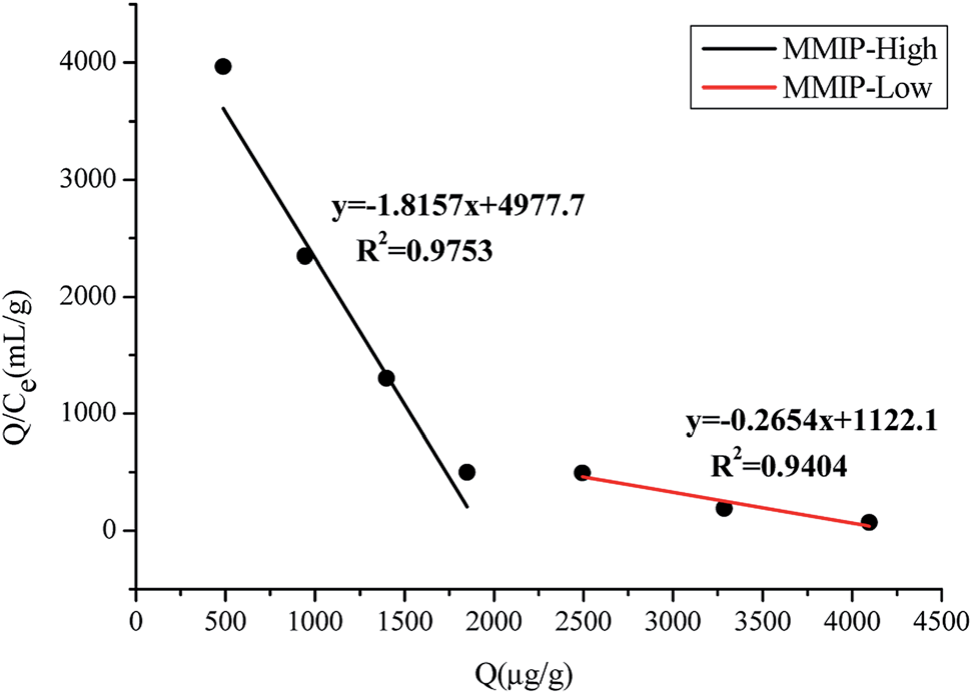
Scatchard equation of MMIP.

The Scatchard equation was calculated and displayed in Fig. 7. It could be concluded that the MMIP provided two different binding sites for adsorption: high and low binding sites. Calculated from the slopes and intercepts of the fitted lines, the *K*_d_ values were 0.55 and 3.77 μg mL^−1^, and the *Q*_max_ values were 2737.74 and 4226.55 μg g^−1^, respectively.

The adsorption capacity for non-structure analogues (chloramphenicol) and pyridine carboxylic acid (picloram, fluroxypyr and clopyralid) that possessed structural similarities to MMIP was evaluated to determine the selectivity of the MMIP. The results illustrated that the MMIP has almost no adsorption capacity to chloramphenicol due to the lack of imprinted sites specific for compounds with different molecular stereochemistry. The developed MMIP obviously exhibited a high binding affinity for aminopyralid and picloram, while other structurally similar compounds (fluroxypyr, and clopyralid) showed less adsorption affinity as shown in Fig. S3.†

The eluted MMIP was reused for five consecutive adsorption–desorption cycles. The results showed that *t* the adsorption capacity of aminopyralid on MMIP has little change after seven cycle's regeneration. This finding indicates that MMIP can maintain its excellent affinity and reusability after multiple adsorptions–desorption cycles.

### 3.3. Optimization of the MI-MDSPE method

The MMIP was used in dispersive SPE and the conditions of the MI-MDSPE method were optimized.

#### 3.3.1 Effects of the extraction solution on the recovery

**Fig. 8:**
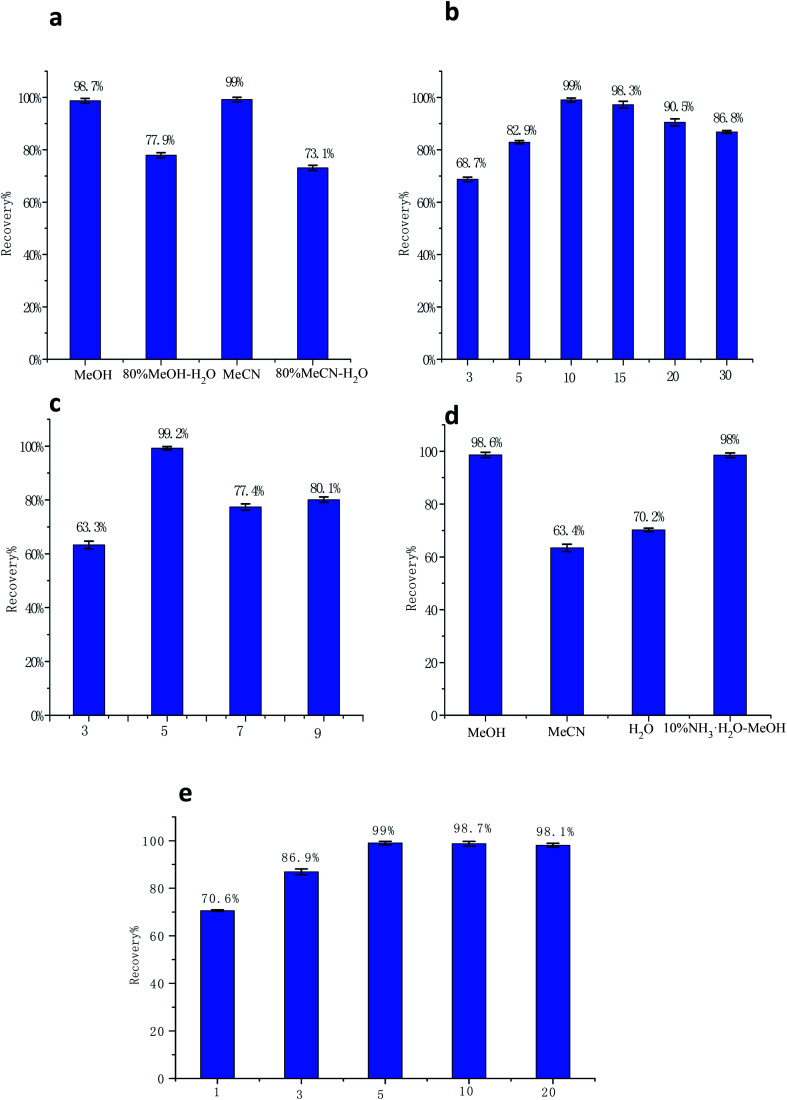
Effect of different extracts (a), extraction time (b), pH (c), eluent (d), and elution time (e) on the recovery of aminopyralid in milk samples.

To identify the effect of the extraction solution on the recovery of the target compound; methanol, acetonitrile and their aqueous solutions were tested. As shown in Fig. 8a, the recoveries were higher using methanol and acetonitrile as the extraction solution; however, the presence of water in the solvent would cause poor recoveries. Therefore, in the preparation of milk samples, water must be removed. Considering that the analyte was extracted from milk with acetonitrile, we have chosen acetonitrile as an extraction solvent.

#### 3.3.2 Effects of the extraction time on the recovery

Different shaking times from 3 to 30 min were tested to obtain the optimum time for mixing the sorbent and solution. As shown in Fig. 8b, the recovery increased with an increase in extraction time up to 10 min and threafter slowly decreased with extraction time. Therefore, the optimum extraction time was set at 10 min.

#### 3.3.3 Effects of the pH on the recovery

The solution pH could substantially affect the stability of polymer structures and existing forms of analytes.^24,28^ In this work, sample pH values ranging from 3 to 9 were studied. As shown in Fig. 8c, the recovery reached 99.2% when the pH was ≤5 and then decreased with further increases in pH. The strong interaction between the analyte and MMIP in weakly acidic solution would maintain the stability of the bond between these two species. However, this bond was destroyed by both alkaline and strongly acidic solution. Thus, pH 5 was selected for subsequent experiments.

#### 3.3.4 Effects of the eluent and elution time on the recovery

After determining the optimal extraction solution, extraction time, and pH, the elution solution was optimized. Three millilitres of organic solvent, including methanol, acetonitrile, water, and 10% ammonium hydroxide-methanol were investigated for coeluting the analyte (Fig. 8d). The results showed that methanol and 10% ammonium hydroxide-methanol have good elution ability, however, 10% ammonium hydroxide-methanol was not chosen as the elution solution due to the long nitrogen blowing time and poor environmental protection of ammonium hydroxide. Subsequently, the elution time of the analyte from the MMIP was optimized in the range of 1 to 20 min. As shown in Fig. 8e, the analyte was completely removed from the sorbent at 5 min.

### 3.4. Validation of the MI-MDSPE-LC-MS/MS method

To validate the MI-MDSPE-LC-MS/MS method, analytical performance characteristics such as sensitivity, detection limits, accuracy, precision, and interferences were evaluated.

The linearity of the method was tested by adding different concentrations (1, 5, 10, 20, 50, and 100 μg L^−1^) of aminopyralid to blank milk samples. Good linearities were obtained for analyte with correlation coefficients (*R*^2^) higher than 0.9972. The LOD (S/N = 3) and LOQ (S/N = 10) for aminopyralid were 0.231 μg kg^−1^ and 0.77 μg kg^−1^, respectively, which are sufficient for determination of aminopyralid in milk.

The precision of the developed method was investigated by spiking blank milk samples with aminopyralid at three different concentrations (10, 20, and 50 μg kg^−1^) in triplicates. As complied in Table 1, the recovery rates were ranged from 83.3–90% with relative standard deviations (RSDs) < 12.6%; the finding which indicates that the MI-MDSPE-LC-MS/MS is a reliable method. Compared with other methods, MI-MDSPE-LC-MS/MS showed great advantages, especially in terms of LOD (Table 2). Therefore, MMIP has a great potentiality for practical application in detecting aminopyralid in milk samples.

**Table 1:** Recoveries, RSD, LOD, and LOQ of aminopyralid in milk samples (*n* = 3)

Analyte	Spiking level (μg L^−1^)	Recovery (%)	RSD (%)	LOD (μg kg^−1^)	LOQ (μg kg^−1^)
Aminopyralid	10	90	12.6	0.231	0.77
	20	87.4	9.8		
	50	83.3	10.2		

**Table 2:** Comparison between different detection methods of aminopyralid

Method	Pretreatment methods	Sample	Recovery (%)	LOD (μg kg^−1^)	LOQ (μg kg^−1^)	Analysis time	Ref.
LC-MS/MS	QuEChERS^a^	Cucumber, eggplant, tomato, apple, grape	70.0–109.4%	10–90	21–36	1.5 h	Tian *et al.*, 2012
GC-ECD	SPE^b^	Forage grass, hay, soil	80.0–104%	10–20	20–50	2 h	Li *et al.*, 2018
LC-MS/MS	SPE^b^	Barley	76.5–88.4%	10	50	2 h	Zhang *et al.*, 2014
LC-MS/MS	MI-MDSPE^c^	Milk	83.3–90%	0.231	0.77	1 h	This work

a Quick, easy, cheap, effective, rugged, and safe method.

b Solid-phase extraction.

c Dummy molecular imprinting-magnetic dispersive solid-phase extraction.

## 4. Conclusions

In this study, MMIPs were synthesized and used as adsorbents in dispersive SPE to adsorb aminopyralid in milk samples prior to determination by LC-MS/MS. MMIPs with a core–shell structure were synthesized by coating dummy molecular template (picloram), functional monomers (4-VP) and cross-linking agent (TRIM) on the surface of Fe_3_O_4_@SiO_2_ modified with amino groups in methanol. Adsorption tests indicated that the MMIP specifically adsorbs aminopyralid. Furthermore, the MMIP was employed in dispersive SPEand the results showed that it can be used as an extraction material to detect aminopyralid in milk.

## Conflicts of interest

Authors have declared no conflicts of interest.

## Supplementary Material

RA-009-C9RA05782J-s001
