# Supplementary material for: Magnetic molecularly imprinted polymers for the detection of aminopyralid in milk using dispersive solid-phase extraction
Source: RSC Adv. 2019 Sep 23;9(51):29998–30006. doi: 10.1039/c9ra05782j (PMC9072149; doi:10.1039/c9ra05782j)
Supplement: RA-009-C9RA05782J-s001 [file RA-009-C9RA05782J-s001.pdf]

Electronic Supplementary Material (ESI) for RSC Advances.
This journal is © The Royal Society of Chemistry 2019

Supporting Information

Magnetic molecularly imprinted polymers for the detection of aminopyralid in milk using dispersive solid-phase extraction

Yahui He[1,2*], Sijia Tan[2], A. M. Abd EI-Aty[3,4], Ahmet Hacımüftüoğlu[4], Yongxin She[2]**

[1]*Beijing Technology and Business University, 100048, P.R. China*

[2]*Institute of Quality Standards & Testing Technology for Agro-Products, Chinese Academy of Agricultural Sciences, Beijing 100081, P.R. China*

[3]*Department of Pharmacology, Faculty of Veterinary Medicine, Cairo University, 12211-Giza, Egypt*

[4]*Department of Medical Pharmacology, Medical Faculty, Ataturk University, 25240-Erzurum, Turkey*

Corresponding authors:

**(Y.H.) Tel: +86 01068985456; Fax: +86 010 68985456. E-mail: hyh@btbu.edu.cn*

***(Y.S.) Tel: +86 010 82106513; Fax: +86 010 82106567. E-mail: sheyongxin@caas.cn*

Table S1. The design and optimization of the polymerization system

Template	Functional monomer	Cross-linker	Porogen	Molar ratio	Adsorption quantity ($\mu g \cdot g^{-1}$)
				1:2:6	1297.5
aminopyralid	4-VP	TRIM	Methanol	1:4:6	1348.2
				1:6:6	1403.1
				1:2:10	1228.4
aminopyralid	4-VP	TRIM	Methanol	1:4:10	1232.1
				1:6:10	1303.7
				1:2:20	892.3
aminopyralid	4-VP	EGDMA	Methanol	1:4:20	942.5
				1:6:20	992.6
				1:2:6	863.2
aminopyralid	4-VP	TRIM	Dichloromethane	1:4:6	720.1
				1:6:6	792.4
				1:2:10	817.5
aminopyralid	4-VP	TRIM	Dichloromethane	1:4:10	735.2
				1:6:10	808.3
				1:2:6	1001.5
aminopyralid	MAA	TRIM	Methanol	1:4:6	1209.2
				1:6:6	1159.9
				1:2:10	953.9
aminopyralid	MAA	TRIM	Methanol	1:4:10	1025.0
				1:6:10	939.1
				1:2:20	704.2
aminopyralid	MAA	EGDMA	Methanol	1:4:20	967.3
				1:6:20	883.4

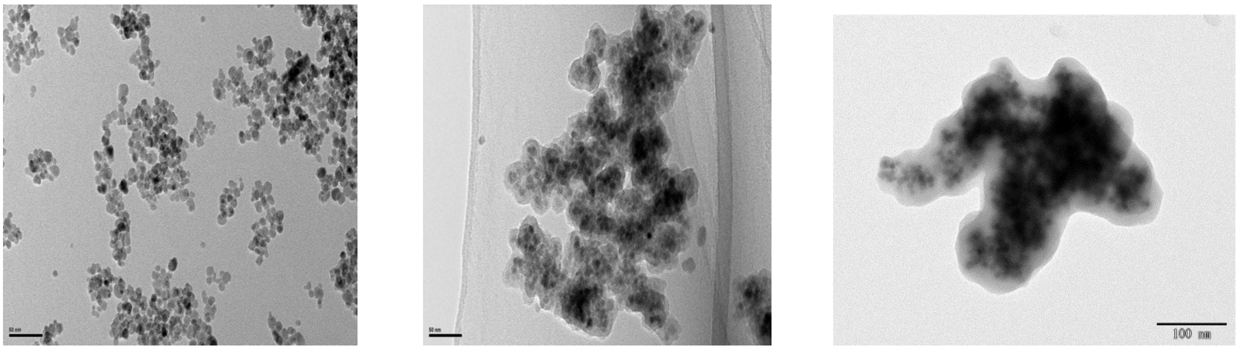

a b c

Fig.S1 the TEM images of Fe3O4(a), Fe3O4@SiO2-COOH(b), and Fe3O4@SiO2-NH2@NIP(c).

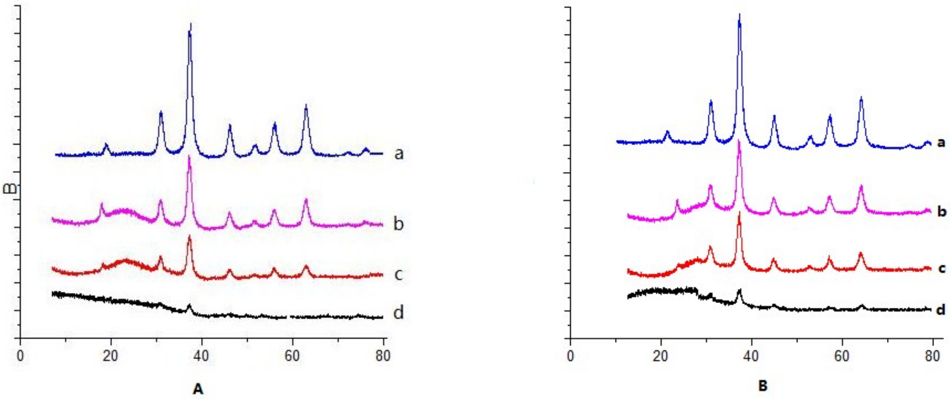

Fig. S2. A. The XRD patterns of Fe_3O_4 nanoparticles(a), $Fe_3O_4@SiO_2$ (b) , $Fe_3O_4@SiO_2$–COOH (c), $Fe_3O_4@SiO_2$–COOH@MIP (d)

B. The XRD patterns of Fe_3O_4 nanoparticles(a), $Fe_3O_4@SiO_2$ (b) , $Fe_3O_4@SiO_2$–NH_2 (c), $Fe_3O_4@SiO_2$–NH_2@MIP (d)

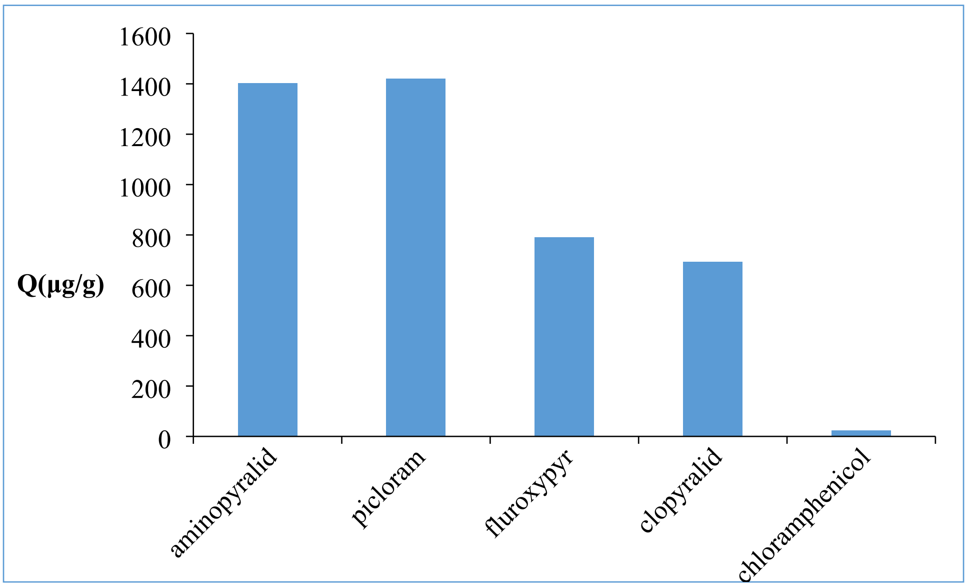

Fig.S3. the selectivity of MMIP
